# Sleep in Offspring of Parents With Mood Disorders

**DOI:** 10.3389/fpsyt.2019.00225

**Published:** 2019-04-08

**Authors:** Delainey L. Wescott, Jessica Morash-Conway, Alyson Zwicker, Jill Cumby, Rudolf Uher, Benjamin Rusak

**Affiliations:** ^1^Department of Psychology and Neuroscience, Dalhousie University, Halifax, NS, Canada; ^2^Department of Psychiatry, Dalhousie University, Halifax, NS, Canada; ^3^Nova Scotia Health Authority, Halifax, NS, Canada; ^4^Department of Pathology, Dalhousie University, Halifax, NS, Canada

**Keywords:** sleep, severe mental illness, major depressive disorder, bipolar disorder, actigraphy, high-risk offspring, cohort study

## Abstract

**Background:** Sleep problems in childhood are an early predictor of mood disorders among individuals at high familial risk. However, the majority of the research has focused on sleep disturbances in already diagnosed individuals and has largely neglected investigating potential differences between weeknight and weekend sleep in high-risk offspring. This study examined sleep parameters in offspring of parents with major depressive disorder or bipolar disorder during both weeknights and weekends.

**Methods:** We used actigraphy, sleep diaries, and questionnaires to measure several sleep characteristics in 73 offspring aged 4–19 years: 23 offspring of a parent with major depressive disorder, 22 offspring of a parent with bipolar disorder, and 28 control offspring.

**Results:** Offspring of parents with major depressive disorder slept, on average, 26 min more than control offspring on weeknights (95% confidence interval, 3 to 49 min, *p* = 0.027). Offspring of parents with bipolar disorder slept, on average, 27 min more on weekends than on weeknights compared to controls, resulting in a significant family history × weekend interaction (95% confidence interval, 7 to 47 min, *p* = 0.008).

**Conclusions:** Sleep patterns in children and adolescents were related to the psychiatric diagnosis of their parent(s). Future follow-up of these results may clarify the relations between early sleep differences and the risk of developing mood disorders in individuals at high familial risk.

## Introduction

Sleep disturbances are core symptoms of mood disorders including major depressive disorder and bipolar disorder (1). Additionally, sleep problems have been associated with more severe symptoms, greater functional impairment, and increased risk for relapse among individuals with mood disorders (2). Over 40% of children and youth experience sleep disturbances during development (3). Early sleep disturbances have been shown to predict depressive symptoms (4, 5), manic episodes (6, 7), and psychotic-like experiences (8). However, there have been some inconsistencies in the reported relationship between sleep and psychopathology (9). The persistence of sleep problems may represent a key factor in the true relationship between sleep disturbance in childhood and later mental illness. Many sleep problems resolve during development, but those that persist predict symptoms of depression, anxiety, and externalizing behaviors into adulthood (10). These findings highlight the importance of treating sleep disturbances as a contributing factor to, and not just a symptom of, mood disorders, and emphasize the need for longitudinal investigations of these relationships.

Many previous studies examining the relationship between sleep disturbances and mood disorders focused on individuals who had already been diagnosed (11). There has been less research examining sleep disturbances among children and youth at high familial risk of mood disorders. This gap in the literature is surprising given that offspring of parents with a mood disorder have a 1 in 3 chance of developing a mental illness themselves (12).

Mood disorders typically have an onset in the second or third decade of life; however, they are often preceded by earlier manifestations, which have been referred to as antecedents (13), defined as conditions that precede the onset of mood disorders by at least several years (14). Longitudinal high-risk studies suggest that sleep disturbances likely represent an antecedent to mood disorders (14–16). However, there is limited information on early sleep disturbances among offspring of parents with mood disorders. Offspring of mothers with postpartum depression are more likely than control offspring to have sleep problems (17) and to develop depression themselves (18). Consistent with these findings, there is evidence that sleep disturbances are prevalent among offspring of a parent with bipolar disorder (7, 19, 20).

Previous research on sleep in high-risk offspring has relied largely on self-reported measures of sleep (7, 14) despite the known benefits of combining self-report and objective measures (21). Actigraphy is an objective, indirect measure of sleep that uses accelerometer-based devices to estimate sleep parameters (22) and provides an ecologically valid representation of typical sleep patterns in a participant’s natural environment (23). Studies that have used objective sleep measures have typically not reported sleep data from weeknight and weekends separately, despite recommendations to do so (24, 25). Discrepancies in sleep patterns between weeknights and weekends are prevalent during adolescence. These discrepancies are linked to emotional disturbances and have been shown to be independently associated with self-harm among adolescents (26). Additionally, sleep duration on free days (e.g., weekends or vacation) has been shown to be more heritable than sleep duration on sleep-constrained days (e.g., weeknights preceding school days) (27), and variability in weeknight and weekend sleep is a common sleep pattern observed in individuals with bipolar disorder (14). This reinforces the importance of evaluating these sleep parameters on weeknights and weekends separately among offspring at high familial risk of mood disorders.

The current study used actigraphy, sleep diaries, and self-report sleep questionnaires to examine sleep among offspring of parents with mood disorders during both weeknights and weekends. Sleep period length, total sleep time (TST), sleep onset latency (SOL), and wake after sleep onset (WASO) were compared between offspring at high familial risk of mood disorders and offspring of control parents. We hypothesized that children and youth at familial risk of developing a mood disorder would have more disturbed sleep than offspring of controls, as indicated by longer SOLs, increased WASO, and larger weeknight–weekend discrepancies in TST.

## Materials and Methods

### Sample Description

We measured sleep in children and youth aged 4–19 years who participate in the Families Overcoming Risks and Building Opportunities for Well-being (FORBOW) cohort, which includes offspring of parents with severe mental illness and compares them to controls with no known familial risk (28).

Offspring of parents with mood disorders were recruited from February 2015 to June 2018 through their parents’ contact with mental health services in Nova Scotia, Canada. Offspring of control parents matched for age and socioeconomic status were recruited through local school boards. In this study, we included offspring with a parent diagnosed with major depressive disorder or bipolar disorder, as well as offspring of control parents. Participants and at least one parent were required to have the capacity to read, write, and speak English. We excluded youth with severe intellectual disability or autism spectrum disorders. No exclusions were made based on sex, ethnicity, living arrangements, medication, or other psychopathology.

### Parent Assessment

Diagnoses of mental disorders according to the Diagnostic and Statistical Manual of Mental Disorders, Fourth Edition (DSM-IV) and DSM-5 were established using the Schedule for Affective Disorders and Schizophrenia (SADS for DSM-IV) and the Structured Clinical Interview for DSM-5 Disorders (SCID-5) (29). Diagnoses were confirmed in consensus meetings with a psychiatrist blind to offspring psychopathology. Offspring were placed in family history groups based on their biological parent’s diagnosis. In cases where offspring had two parents with different diagnoses, they were placed in the family history group based on the DSM-5 diagnostic hierarchy. Thus, offspring with at least one parent with bipolar disorder were placed in the bipolar disorder family history group, because a bipolar disorder diagnosis is considered definite. This group included a subset of offspring who also had a parent with a diagnosis of major depressive disorder, which are referred to here as dual-risk offspring (see the section Results).

### Actigraphy and Sleep Diary

Participants wore Micro Motionlogger actigraphs (Ambulatory Monitoring Inc., Ardsley NY) on their nondominant wrist every day for a 2-week period, beginning on a Sunday. They were instructed to remove the actigraph during any water-related activities and while participating in sports. Communication check-ins were made approximately once per week to ensure that there were no difficulties with the protocol or with the actigraphs. Each night was analyzed independently. Additionally, weekdays and weeknights were analyzed separately. Weeknights were defined as Sunday through Thursday nights, and weekends were defined as Friday and Saturday nights.

Data were sampled in 60-s epochs, collected in Zero-Crossing Mode, and analyzed with Action W2.7 using the Cole–Kripke scoring algorithm (30). The Cole–Kripke algorithm has been validated in adult and child populations and has been found to be more accurate in estimating TST than the alternative Sadeh algorithm (24, 31). From the actigraphy data, we extracted information about the sleep period, TST, SOL, and WASO separately to determine the impact of familial history on each characteristic of sleep. Sleep period was defined as the duration between sleep onset (time fell asleep) and sleep end (morning wake time). TST was the number of actual sleep minutes during the sleep period (i.e., excluding intervening wake episodes).

In accordance with best practice parameters for actigraphy (32, 33), sleep diaries were used to clarify any ambiguous actigraphy data and provided a timeline for when and how long the participants wore the actigraphs. Participants filled out a sleep diary every day during the 2-week period as recommended by Buysse et al. (21). Participants older than 15 completed a self-report sleep diary, and parents completed a parent report sleep diary for participants younger than 15. Information collected in the sleep diaries included nightly bedtimes, time required to fall asleep, morning wake times, and daytime naps.

### Self-Reported Measures of Sleep

#### Children’s Sleep Habits Questionnaire

This 35-item parent report measure assesses sleep problems in children aged 4–14 years (34). This questionnaire contains eight subscale scores including bedtime resistance, sleep onset delay, sleep duration, sleep anxiety, night waking, parasomnias, sleep disordered breathing, and daytime sleepiness. The Children’s Sleep Habits Questionnaire (CSHQ) has been validated in clinical and community populations and has been shown to have good internal consistency and test–retest reliability. Scoring of some items was reversed in order to make a higher score consistently indicative of more disturbed sleep (34).

#### School Sleep Habits Survey

This self-report measures sleep/wake habits, sleep patterns during school nights compared to weekends, and daytime functioning of adolescents 15–17 years old (35). The School Sleep Habits Survey (SSHS) consists of 63 questions and has been used in other studies looking at high-risk offspring (7). The SSHS contains three subscales: 1) a depressed mood scale, 2) a sleepiness scale, and 3) a sleep/wake problem behavior scale. The depressed mood scale (36) comprises six items assessing participants’ negative emotions in the 2 weeks prior to completing the questionnaire. This scale has high internal reliability and high test–retest reliability for adolescents (35). The sleepiness scale queries the frequency that participants struggled to stay awake in 10 different situations during the 2 weeks prior to completing the questionnaire. The sleep/wake problem scale comprises 10 items examining the frequency of erratic sleep/wake behaviors in the 2 weeks prior to completing the questionnaire (35).

### Puberty Measurements

During adolescence, there are noticeable changes in sleep–wake behaviors due to a variety of psychosocial and biological factors (37, 38), which are strongly influenced by the stages of puberty (39). To accurately compare sleep parameters between family history groups, we controlled for puberty onset in our analyses. Pubertal onset was determined by the “Growing and Changing Questionnaire” (GCQ) administered annually in the FORBOW cohort at age 8 and older. The GCQ was based on two measures of pubertal status: Pearson’s Puberty Development Scale (PDS) (40) and the Sexual Maturation Scale (SMS) (41, 42). The PDS components of the GCQ asked about body hair, skin changes, growth of facial hair for males, and onset/age of menstruation for females. The SMS section comprises five drawings displaying progressive stages of pubertal development of secondary sexual characteristics ranging from pre-pubertal to post-pubertal. Both the PDS and SMS sections were scored on a 1–5 scale according to Carskadon and Acebo (40) and then averaged to create a composite puberty score. A dichotomous puberty onset score was created to indicate whether each participant was pre- or post-pubertal at the time of sleep assessment. Composite puberty scores ≤2 were scored as pre-pubertal and scores ≥3 were scored as post-pubertal.

### Statistical Analysis

The actigraphy measures of sleep parameters (sleep period, TST, SOL, and WASO) were calculated using Action W2.7 software. Sleep parameters were analyzed separately for weeknights and weekend nights as recommended by Galland et al. (25).

All statistical procedures were conducted using STATA software package, version 15.1 (43). We fitted mixed-effects generalized linear models to test the effect of family history (no diagnosis, major depressive disorder, or bipolar disorder) on sleep period, TST, SOL, and WASO. We included age, sex, pubertal onset, and current diagnosis of Attention Deficit Hyperactivity Disorder (ADHD) as fixed covariates in each model. We accounted for the non-independence of observations from related individuals and from repeated measures from the same individual by including family and individual identifiers as random effects in the models. We conducted sensitivity analyses controlling for stimulant use, cannabis use, and a current diagnosis of anxiety. We also conducted sensitivity analyses removing offspring with current diagnoses of bipolar disorder (*n* = 1) or major depressive disorder (*n* = 2). We assessed the subscales for the CSHQ and the SSHS for internal consistency using Cronbach’s α-coefficients. We used mixed-effects generalized linear models with the same fixed covariates and random effects described above for comparisons between family history groups.

## Results

### Sample Description

A total of 86 participants completed the study. Actigraphy data from 13 participants were not included in the analysis due to loss of the actigraph before retrieving the data (*n* = 3), long periods of time without wearing the actigraph (*n* = 2), or corrupted actigraphy data (*n* = 8). Of the remaining 73 participants (actigraphy completion rate 86.2%), 23 were offspring of a parent with major depressive disorder (9 of whom had both parents with major depressive disorder), 22 were offspring of a parent with bipolar disorder (9 of whom had one parent with bipolar disorder and one parent with major depressive disorder), and 28 were offspring of controls (**Table 1**). There were 45 pre-pubertal and 28 post-pubertal participants. There were 20 offspring with a current diagnosis: 9 with ADHD, 8 with anxiety, 2 with major depressive disorder, and 1 with bipolar disorder.

**Table 1:** Demographic and clinical information for the sample.

	Control offspring (*n* = 28)	Offspring of MDD (*n* = 23)	Offspring of BD (*n* = 22)	Total (*n* = 73)
Age, mean (SD)	11.01 (2.97)	11.44 (3.44)	12.36 (3.32) *11.99 (3.86)* ^1^	11.55 (3.23)
Female, *n* (%)	8 (29%)	11 (48%)	8 (36%) *4 (44%)* ^1^	27 (37%)

1 Demographic and clinical information for dual-risk offspring of one parent with bipolar disorder and one parent with major depressive disorder (n = 9).

### Questionnaire Scores

Among the participants who completed all questionnaires (total completion rate, 75.3%), offspring of a parent with bipolar disorder scored significantly higher on the sleep/wake problems scale of the SSHS compared to controls (β = 8.79, 95% CI: 0.83, 16.75; *p* = 0.030). There were no significant differences among offspring from the three family history groups for the depressed mood subscale or the sleepiness subscale calculated from the SSHS (see **Table 2**). There were no group differences for the eight subscales of the CSHQ or the total CSHQ score (see **Table 2**).

**Table 2:** Mean and standard deviations of parent-reported Children’s Sleep Habits Questionnaire (CSHQ) total score and subscales by family history group for offspring aged 4–14. Mean and standard deviations of self-reported School Sleep Habits Survey (SSHS) subscales by family history group for offspring aged 15–17.

	Offspring of controls	Offspring of MDD	Offspring of BD
Children’s Sleep Habits Questionnaire^1^			
Participants (*n*)	17	14	9
	Mean (SD)	Mean (SD)	Mean (SD)
Subscale Item^2^			
Bedtime resistance	8.50 (2.89)	6.64 (0.84)	7.89 (2.15)
Sleep onset delay	1.56 (0.71)	1.36 (0.63)	1.78 (0.67)
Sleep duration	4.47 (1.74)	3.29 (0.47)	4.00 (1.58)
Sleep anxiety	5.50 (2.20)	4.79 (1.48)	6.00 (2.24)
Night wakings	3.50 (0.92)	3.21 (0.58)	3.56 (0.76)
Parasomnias	7.28 (1.23)	6.36 (0.63)	7.89 (0.92)
Sleep disordered breathing	1.12 (0.33)	1.43 (0.76)	1.11 (0.33)
Daytime sleepiness	8.50 (2.92)	9.93 (2.56)	9.78 (3.93)
Total score	40.44 (9.79)	37.00 (2.63)	42.00 (7.38)
			
School Sleep Habits Survey^3^			
Participants (*n*)	4	3	5^4^
	Mean (SD)	Mean (SD)	Mean (SD)
Subscale item			
Depressed mood scale	6.90 (1.80)	7.00 (0.00)	11.00 (3.74)
Sleepiness scale	11.00 (2.00)	12.00 (3.46)	12.80 (2.68)
Sleep/wake problems behavior scale	19.29 (3.15)	23.57 (5.96)	28.96*(6.77)

1 Parents of 48 offspring between ages 4 and 14 completed the CSHQ (completion rate, 70.6%).

2 The names of the items in this table are those used in the CSHQ.

3 Twelve offspring between ages 15 and 17 completed the SSHS (completion rate, 92.3%).

4 Two offspring in the bipolar family history group were dual-risk offspring who also had a parent with major depressive disorder.

*p < 0.05.

### Sleep Diary Results

As recommended by Meltzer et al (24), we calculated the average self- or parent-reported bedtime and waketime for each family history group (see **Table 3**). We also calculated the average sleep period as the time between bedtime and wake time. There were no significant differences between family history groups in bedtimes, waketimes, or sleep periods on either weeknights or weekends.

**Table 3:** Sleep parameters derived from 14 days of actigraphy data by family history group.

	Control offspring (*n* = 28)		Offspring of MDD (*n* = 23)		Offspring of BD only (*n* = 13)		Offspring of BD and MDD (*n* = 9)		All Offspring of BD (*n* = 22)	
	Mean	SD (min)	Mean	SD (min)	Mean	SD (min)	Mean	SD (min)	Mean	SD (min)
Weekdays										
Sleep onset (HH : MM)	22:06	117.61	22:05	108.63	22:17	118.08	22:56	155.55	22:33	82.95
Sleep end (HH : MM)	7:21	60.03	7:33	78.45	07:22	94.02	8:05	131.04	7:40	69.69
										
*Variables of Interest*										
Sleep period (min)	555.36	64.81	572.08*	68.00	559.73	66.14	534.41	84.50	538.14	74.96
TST (min)	488.87	64.26	510.53*	61.34	496.03	61.51	473.17	91.61	477.83	72.01
SOL (min)	11.17	12.25	8.35	6.88	9.72	11.53	10.00	11.10	8.8	14.39
WASO (min)	46.88	34.89	42.74	27.04	44.85	31.13	37.02	25.49	40.27	29.55
										
Weekends										
Sleep onset (HH : MM)	22:34	138.68	22:39	116.15	22:44	120.35	23:01	141.21	22:54	83.95
Sleep end (HH : MM)	7:50	123.26	7:59	97.23	8:09	158.54	8:54	129.43	8:28	68.52
										
*Variables of Interest*										
Sleep period (min)	557.05	80.88	561.25	90.59	557.85	80.61	594.03*	66.19	577.89*	67.19
TST (min)	484.18	83.58	492.73	93.63	484.65	83.31	519.43	51.56	502.46	64.95
SOL (min)	9.86	10.7	7.67	6.77	9.67	11.04	10.57	8.45	11.67	15.24
WASO (min)	49.42	35.58	44.88	25.77	47.86	31.59	45.20	29.30	47.24	33.13

*p < 0.05.

### Weeknight Actigraphy Results

In contrast to the sleep diary results, we found that the offspring of a parent with major depressive disorder had a significantly longer sleep period (β = 25 min, 95% CI: 4.54, 46.43; *p* = 0.017) and TST than control offspring on weeknights (β = 26 min, 95% CI: 3.01, 49.46; *p* = 0.027; see **Table 3**). We looked separately at the results from offspring with only one or with two parents with major depressive disorder. Those with two parents with a diagnosis of major depressive disorder (*n* = 9) showed a pattern similar to that of the entire group with a longer sleep period than controls that did not reach threshold for statistical significance (β = 23 min, 95% CI: −5.08, 51.53; *p* = 0.108) and TST (β = 26 min, 95% CI: −5.15, 56.54; *p* = 0.103). Offspring of only one parent with major depressive disorder (*n* = 14) showed a significantly longer sleep period (β = 26.95 min, 95% CI: 2.52, 51.36; *p* = 0.031) and a similar, but not statistically significant, increase in TST (β = 26.61 min, 95% CI: −0.72, 53.94; *p* = 0.056).

**Figure 1:**
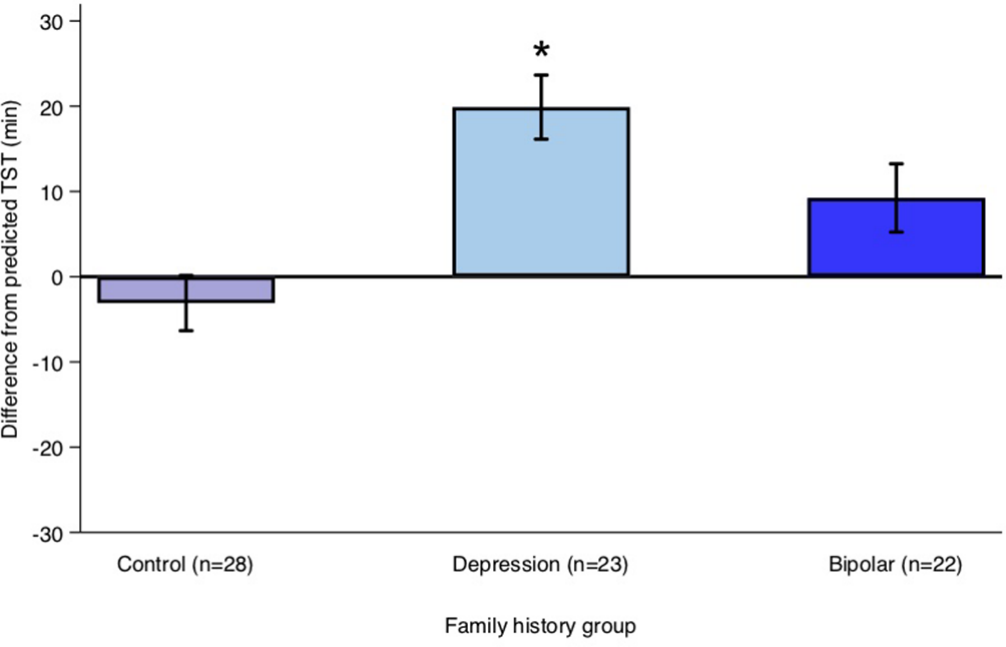
Average difference between actual total sleep time (TST) and predicted TST on weeknights for each family history group. Predicted TST was calculated using a linear prediction from a model predicting TST using age, sex, and pubertal onset for controls during weeknights. Error bars represent the standard error of the mean. *Note:* *p < 0.05.

Offspring of a parent with bipolar disorder did not differ significantly from control offspring in their sleep period (β = −3 min, 95% CI: −23.99, 17.95; *p* = 0.777) or TST (β = −0.79 min, 95% CI: −23.86, 22.28; *p* = 0.946) on weeknights. Average differences between TST calculated from actigraphy and “predicted” TST on weeknights for each family history group are shown in **Figure 1**. Predicted TST was calculated using a linear prediction from a model based on age, sex, and pubertal status for controls during weeknights.

### Weekend Actigraphy Results

Offspring of a parent with bipolar disorder showed longer, but not statistically significantly different, sleep periods than controls (β = 23 min, 95% CI: −1.10, 49.04; *p* = 0.061) and TST (β = 20 min, 95% CI: −8.59, 48.79; *p* = 0.170; see **Table 3**). The “dual-risk” subgroup of this group (offspring of one parent with bipolar disorder and one with major depressive disorder) showed a significantly longer sleep period compared to controls on weekend nights (β = 35 min, 95% CI: 2.55, 68.71; *p* = 0.035). Offspring of a parent with major depressive disorder did not differ significantly from controls in their sleep period (β = 5 min, 95% CI: −18.94, 28.95; *p* = 0.682) or TST (β = 8 min, 95% CI: −19.93, 35.74; *p* = 0.578) on weekends.

### Family History by Weekend Interaction Actigraphy Results

**Figure 2:**
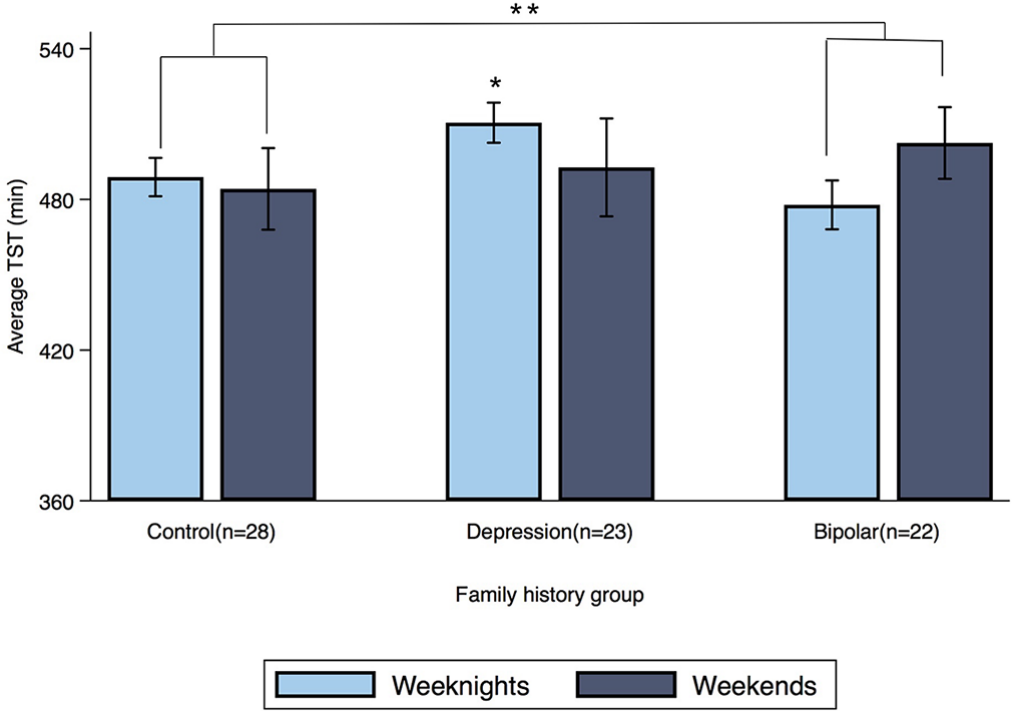
Average TST for weeknights and weekends by family history group. Error bars represent the standard error of the mean. *Note:* *p < 0.05, **p < 0.01.

Offspring of a parent with bipolar disorder showed a significant family history by weekend interaction for TST (β = 27 min, 95% CI: 7.16, 47.49; *p* = 0.008) compared to controls (see **Figure 2**). When this family history group was separated into its two subgroups, dual-risk offspring (*n* = 9; offspring of one parent with bipolar disorder and one with major depressive disorder) showed a family history by weekend interaction for TST (β = 49 min, 95% CI: 22.33, 75.21; *p* < 0.001), but offspring with one parent with bipolar disorder and a healthy co-parent (*n* = 13) did not (β = 11 min, 95% CI: −12.12, 35.31; *p* = 0.338).

The family history by weekend interaction was also significant for sleep period in offspring of a parent with bipolar disorder (β = 36 min, 95% CI: 15.76, 57.07; *p* = 0.001). Dual-risk offspring showed a family history by weekend interaction for sleep period (β = 56 min, 95% CI: 28.73, 82.69; *p* < 0.001) compared to controls, but offspring with one parent with bipolar disorder and a healthy co-parent showed only a weaker tendency toward the same effect (β = 22 min, 95% CI: −1.96, 46.49; *p* = 0.072). Offspring of a parent with major depressive disorder did not show a family history by weekend interaction for either TST (β = −13 min, 95% CI: −32.80, 6.45; *p* = 0.188) or sleep period (β = −11 min, 95% CI: −31.83, 8.38; *p* = 0.253) compared to control offspring.

### Sensitivity Analyses

The significant effects remained unchanged in sensitivity analyses that excluded participants who used stimulant medication or cannabis as determined by self-report measures in the FORBOW study. The significant effects also remained when removing offspring with a current diagnosis of bipolar disorder, major depressive disorder, or anxiety. When offspring with anxiety were removed from the sample, offspring of a parent with bipolar disorder had a significantly longer sleep period on weekends (β = 37 min, 95% CI: 11.71, 62.70; *p* = 0.004). There were no other significant differences in actigraphy results between the groups.

## Discussion

Sleep disturbances, including hypersomnia, insomnia, and irregular sleep patterns, are associated with increased risk of mood disorders (7, 44, 45), as are sleep features such as higher Rapid Eye Movement sleep density in high-risk individuals (46). Using actigraphy, we found differences in sleep patterns and durations between offspring of parents with mood disorders and control offspring. We found longer sleep periods and TST on weeknights among offspring of a parent with major depressive disorder and weeknight–weekend differences from controls in sleep duration among offspring of a parent with bipolar disorder and a parent with major depressive disorder. It is noteworthy that these effects seen during 2 weeks of actigraphic recording were not matched by any significant group differences in sleep diaries, nor according to the CSHQ. However, we did find significantly more sleep/wake problems in offspring of a parent with bipolar disorder compared to controls as reported on the SSHS.

The increased sleep duration on weekends compared to weeknights among offspring of parents with bipolar disorder is consistent with earlier results based on interviews, which found that at-risk offspring had high rates of sleep disorders (14). Among those at risk for bipolar disorder, a small pilot study suggested that an intervention aimed at stabilizing sleep–wake patterns over the week may be beneficial in reducing sleep disturbances (47).

When we analyzed data separately for the dual-risk offspring (one parent with bipolar disorder and one with major depressive disorder) and the offspring of only one parent with bipolar disorder, we found that the dual-risk offspring were driving these weeknight–weekend differences in TST. It is unclear whether this pattern is specific to this diagnostic configuration or reflects additional familial loading of risk resulting from having two parents with any mood disorder diagnosis. However, our results comparing offspring with one or two parents with major depressive disorder do not support a generalized risk of having both parents with any mood disorder diagnosis. Rather, they highlight a potential specific risk associated with having one parent with bipolar disorder and one parent with major depressive disorder. In this context, the fact that major depressive disorder is sometimes re-diagnosed as bipolar disorder allows for the possibility that some of these families will include two parents with bipolar disorder. Future follow-ups may permit assessment of this possibility. This result suggests that while altered weeknight–weekend sleep patterns may be an early indicator of increased risk for development of bipolar disorder, those who have two parents with this specific diagnostic configuration may be at greater risk.

Offspring of parents with major depression had longer sleep periods and TST during weeknights than controls. Most treatments for sleep disturbances associated with major depressive disorder to date have focused on treating insomnia (2). Our results suggest that hypersomnia may be an early indicator of increased risk for depression in some children and could be a relevant target for future sleep-based interventions for individuals at high familial risk.

### Strengths and Limitations

Our study benefited from the inclusion of offspring at high familial risk for multiple forms of mood disorders across a broad age range and the use of an objective measure of sleep quality in a relatively large sample of high-risk youth. Actigraphic recording for 2 weeks allowed us to assess weeknight–weekend differences, which proved to be informative. Questionnaire data, which are known to detect sleep abnormalities in clinical populations, did not reproduce the results found with actigraphy in this high-risk population, with the exception of identifying increased sleep problems in offspring of a parent with bipolar disorder. These findings suggest caution in interpreting results of questionnaires and short-term actigraphy data in at-risk children.

Several factors in this study limit the strength of our conclusions. Because of the number of participants in each group, we could not compare results among groups at different ages across the study sample. We did, however, control for both age and pubertal status in our statistical analyses. Because of power limits, we also reported our results as nominal significance levels. It should be noted that there were only nine participants in the dual-risk group; thus, the results for this subgroup should be considered preliminary. Overall, our results suggest that familial risk has an effect on offspring’s sleep patterns; however, it is not possible to distinguish whether parentally contributed genes, the impact of parental illness on the family/offspring, or an interaction of the two contributes to these differences, especially in dual-risk offspring.

The FORBOW study is designed as a cohort with ongoing recruitment of children and adolescents. We anticipate adding additional participants to each of the groups, with a special focus on increasing the number of dual-risk offspring. The cohort will be followed for at least a 5-year period, which will allow for determination of the clinical outcomes for these children and adolescents at high risk and for the evaluation of the predictive power of early sleep–wake characteristics in the development of mood disorders. The results should also facilitate development of early sleep interventions tailored to the characteristics of different at-risk groups and the evaluation of their effects on risk of illness.

## Ethics Statement

The protocol was approved by the Research Ethics Board of the Nova Scotia Health Authority, in conformity with the Canadian Tri-Council Policy Statement 2: Ethical Conduct of Research involving Humans (2014). We obtained informed consent from participants who had the capacity to provide it. For participants who did not have the capacity to make an informed decision, a parent or guardian provided written informed consent and the participant provided assent.

## Author Contributions

All authors listed have contributed sufficiently to the project to be included as authors, and all those who are qualified to be authors are listed in the author byline. DW contributed substantially to the conception and design, acquisition of data, and analysis and interpretation of data. DW also drafted the article and gave final approval of the version to be published. JM contributed to the conception and design and to the acquisition of data, revised the manuscript critically for important intellectual content, and gave final approval of the version to be published. AZ contributed to the analysis and interpretation of data, revised the manuscript critically for important intellectual content, and gave final approval of the version to be published. JC contributed to the acquisition of data and gave final approval of the version to be published. RU contributed substantially to the conception and design, acquisition of data, and analysis and interpretation of data; revised the manuscript critically for important intellectual content; and gave final approval of the version to be published. BR contributed substantially to the conception and design and to the analysis and interpretation of data, revised the manuscript critically for important intellectual content, and gave final approval of the version to be published.

## Funding

This project was supported by funding from the Dalhousie Psychiatry Research Fund, Lindsay Family Graduate Scholarship, Canada Research Chairs Program (award number 231397), the Canadian Institutes of Health Research (grant reference numbers 124976, 142738, and 148394), the Brain & Behavior Research Foundation (NARSAD) Independent Investigator Grant 24684, Nova Scotia Health Research Foundation (grants 275319, 1716, and 353892), the Dalhousie Medical Research Foundation, and the Natural Sciences and Engineering Research Council of Canada (grant RGPIN-305).

## Conflict of Interest Statement

The authors declare that the research was conducted in the absence of any commercial or financial relationships that could be construed as a potential conflict of interest.
